# Synthesis and Mechanisms of Scale and Corrosion Inhibition by Ethylenediamine–Benzenesulfonic Acid-Modified Polyaspartic Acid

**DOI:** 10.3390/polym18111301

**Published:** 2026-05-26

**Authors:** Pan Zhang, Yu Han, Xiaogai Lv, Dongyi Li, Linlin Zhao, Shihong Cen, Ying Xu

**Affiliations:** 1 School of Pharmacy and Chemical Engineering, Zhengzhou University of Industrial Technology, Zhengzhou 451150, China; zhangpanzzgy@126.com (P.Z.); shjyjk0204@163.com (X.L.); zzldy12138@126.com (D.L.); 2 School of Chemistry and Molecular Science, Henan University, Kaifeng 475004, China; 104753221132@henu.edu.com; 3 College of Environment and Biological Engineering, Henan University of Engineering, Zhengzhou 451191, China; zhaolin@henu.edu.cn

**Keywords:** modified polyaspartic acid, water treatment agent, scale inhibition, corrosion inhibition, selective antibacterial activity, mechanism

## Abstract

A novel water treatment agent, ethylenediamine–benzenesulfonic acid-modified polyaspartic acid (PASP-S), was controllably synthesized using an amino ring-opening reaction. The controllable synthesis methods, conditions for polymerization degree, and the molecular weight of the new polymer were explored. The structure was characterized using Fourier-transform infrared spectroscopy (FT-IR), ^1^H nuclear magnetic resonance (^1^H-NMR), and gel permeation chromatography (GPC). The scale inhibition, corrosion inhibition, and fluorescence properties of the new polymer, as well as the corresponding mechanisms, were investigated using static scale inhibition tests, electrochemical measurements, X-ray photoelectron spectroscopy (XPS), density functional theory (DFT), and frontier molecular orbital (FMO) theory. The results indicate that PASP-S exhibits strong Ca^2+^ chelation ability and can effectively inhibit CaCO_3_ and CaSO_4_ scaling. At 50 mg/L, the scale inhibition efficiency for Ca_3_(PO_4_)_2_ reaches 99.50%. At 30 mg/L, its corrosion inhibition efficiency is 33.19% higher than that of PASP. Unexpectedly, the polymer shows remarkable selective antibacterial activity. At 100 mg/mL, the inhibition rate against *Escherichia coli* (*E. coli*) is 71%, while no obvious inhibition is observed for *Bacillus cereus*. A good linear relationship is found between fluorescence intensity and concentration. Mechanistic studies demonstrate that PASP-S adsorbs on the scale surface, suppressing crystal growth and distorting crystal morphology. Meanwhile, it forms a protective film on the electrode surface, thus reducing the dissolution and corrosion of carbon steel.

## 1. Introduction

Industrial circulating water is an essential medium for cooling systems in chemical, metallurgical, thermal power, and other industrial sectors and is indispensable for maintaining the stable operation of production equipment and elevating the utilization efficiency of water resources. In industrial circulating water systems, water undergoes long-term cyclic reuse, and owing to temperature rise and salt enrichment, the system readily suffers from scale deposition and pipeline corrosion. Scale accumulation markedly lowers the heat transfer coefficient of heat exchange surfaces, which not only weakens heat exchange efficiency and raises energy consumption and operational costs, but also may trigger pipeline blockage and even system shutdown. In addition, metal corrosion undermines the structural integrity of equipment, brings about leakage hazards and economic losses, and also poses severe risks to production safety [1,2,3].

To prevent and control these hazards, adding scale and corrosion inhibitors to circulating water has become one of the most widely adopted and effective technical strategies in current industrial practices [2,3]. Although a variety of industrial water treatment chemicals have been developed, most still exhibit inherent performance drawbacks. Natural polymer scale inhibitors represented by chitosan, tannin, and cellulose possess desirable characteristics such as excellent environmental friendliness and explicit scale inhibition mechanisms. Nevertheless, their practical application is restricted by high dosage demand, poor thermal stability at elevated temperatures, and low adaptability to complex water quality, thereby limiting their comprehensive application performance [4]. Organophosphonate-based phosphorus-containing scale inhibitors deliver high scale inhibition efficiency and satisfactory corrosion inhibition performance. However, they may induce eutrophication in receiving water bodies after discharge and disrupt aquatic ecological balance. This is inconsistent with increasingly stringent environmental protection regulations, greatly restricting their application in ecologically sensitive regions [5,6].

Polyaspartic acid (PASP) is a typical environmentally friendly water treatment agent. Owing to its excellent biodegradability and superior chelating and dispersing capacity, it has been widely applied in industrial circulating water systems [7,8,9,10,11,12]. To further improve its comprehensive performance, modification research on PASP has attracted extensive academic attention. The existing literature shows that PASP exhibits outstanding scale inhibition performance for CaSO_4_ scale, while its inhibitory effect on CaSO_4_ scale and corrosion inhibition ability are relatively insufficient, failing to satisfy the stringent requirements of industrial circulating water applications [13,14]. To comprehensively enhance the water treatment performance of PASP, functional groups such as hydroxyl, carboxyl, heterocyclic, and sulfonic acid groups or small molecular segments are commonly introduced into its molecular structure to prepare modified PASP derivatives [15].

The sulfonic acid group is an electron-donating group, which can enhance the chelation ability with calcium and magnesium ions and induce lattice distortion of crystals. The π electrons of aromatic rings can form coordination bonds with the empty orbitals of metal ions, thereby improving the compactness of the adsorption film and further strengthening the corrosion inhibition performance [16,17,18,19]. We proposed to modify the side chain of PASP with aromatic substances containing sulfonic acid groups, discuss the influence of sulfonic acid groups on the morphology and crystal type of calcium scale, explore the synergistic effect of aromatic rings and sulfonic acid groups on corrosion inhibition performance, and investigate the influence on colony growth and the fluorescence performance.

## 2. Materials and Methods

### 2.1. Reagents and Instruments

The drugs used in the experiment include the following: polysuccinimide (PSI, AR, Shanghai Macklin Biochemical Technology Co., Ltd., Shanghai, China), ethylenediamine and sodium hydroxide (AR, Foshan Xilong Chemical Co., Ltd., Foshan, China), polysuccinimide (AR, Shanghai Macklin Biochemical Technology Co., Ltd., Shanghai, China, with an average molecular weight of 98.4 g/mol), sodium tetraborate decahydrate (AR, Shanghai Titan Technology Co., Ltd., Shanghai, China), hydrochloric acid (AR, Luoyang Haohua Chemical Reagent Co., Ltd., Luoyang, China), absolute ethanol (AR, Tianjin Fuyu Fine Chemical Co., Ltd., Tianjin, China), ethylenediaminetetraacetic acid disodium salt trihydrate and magnesium sulfate heptahydrate (AR, Anhui Zesheng Technology Co., Ltd., Shanghai, China), L-ascorbic acid (AR, Shanghai Shaoyuan Technology Co., Ltd., Shanghai, China), sodium potassium tartrate (AR, Tianjin Fengchuan Chemical Reagent Technology Co., Ltd., Tianjin, China), hexamethylenetetramine (AR, Shanghai Lingfeng Chemical Reagent Co., Ltd., Shanghai, China), potassium hydroxide (AR, Sinopharm Chemical Reagent Co., Ltd., Shanghai, China), peptone (BG, Shanghai Macklin Biochemical Technology Co., Ltd., Shanghai, China), yeast extract (BG, Shanghai Macklin Biochemical Technology Co., Ltd., Shanghai, China), agar powder (BG, Beijing Solarbio Science & Technology Co., Ltd., Beijing, China), *Escherichia coli* (BG, Shanghai Sangon Biological Engineering Technology & Services Co., Ltd., Shanghai, China), *Staphylococcus aureus* (BG, Shanghai Sangon Biological Engineering Technology & Services Co., Ltd., Shanghai, China), and *Bacillus cereus* (BG, Shanghai Sangon Biological Engineering Technology & Services Co., Ltd., Shanghai, China).

The experimental instruments used in the experiment include the following: mechanical ultrasonic cleaning instrument (KQ-6L, Zhengzhou Guorui Instrument Co., Ltd., Zhengzhou, China), magnetic stirrer (85-2A, Shanghai Sile Instrument Co., Ltd., Shanghai, China), analytical balance with a precision of 0.1 mg (ESI220-4B, Hangzhou Want Balance Co., Ltd., Hangzhou, China), vacuum drying oven (DZF-1050AB, Beijing Zhongxing Weiye Instrument Co., Ltd., Beijing, China), fully digital superconducting nuclear magnetic resonance spectrometer (Bruker AVANCE III, Bruker Corporation, Ettlingen, Germany), X-ray powder diffractometer (Bruker D8 Advance, Bruker Corporation, Karlsruhe, Germany), 3D profilometer (Bruker Contour GT-K, Bruker Nano, Inc., Tucson, AZ, USA), and field emission scanning electron microscope (JSM-7610F, JEOL Ltd., Akishima, Tokyo, Japan).

### 2.2. Structural Characterization

The structure of PASP-S was characterized using a Fourier-transform infrared spectrometer (FTIR-8000, Shimadzu Corporation, Kyoto, Japan) and a nuclear magnetic resonance hydrogen spectrometer (^1^H NMR, Bruker BioSpin GmbH, Ettlingen, Germany). The molecular weight and polydispersity index of PASP-S were determined with gel permeation chromatography (GPC) using a 0.1 M NaNO_3_ aqueous solution as the mobile phase at 1 mL/min flow rate, with PEG as the standard.

### 2.3. Static Scale Inhibition Performance Determination

The scale inhibition performance of PASP-S was evaluated using the calcium carbonate deposition method for determining the scale inhibition performance of water treatment agents (GB/T 16632-2019), the general technical conditions for scale inhibitors in oil fields (SY/T 5673-2020), and the calcium phosphate deposition method for determining the scale inhibition performance of water treatment agents (GB/T 22626-2008) [20,21,22,23]. The scale inhibition performance of PASP-S against calcium carbonate (CaCO_3_), calcium sulfate (CaSO_4_), and calcium phosphate (Ca_3_(PO_4_)_2_) scales was evaluated separately. For CaCO_3_ scale inhibition, the test solution was prepared using calcium chloride (CaCl_2_), sodium bicarbonate (NaHCO_3_), and borax, with mass concentrations of 240 mg·L^−1^ and 732 mg·L^−1^. The reaction was carried out at a constant temperature of 80 °C for 10 h. For CaSO_4_ scale inhibition, the experimental solution contained CaCl_2_, sodium sulfate (Na_2_SO_4_) and borax, which were maintained at 2000 mg·L^−1^ and 4800 mg·L^−1^, respectively, and the test was incubated at 70 °C for 6 h under constant temperature conditions.

After each reaction, the solutions were cooled to room temperature and titrated with a standard EDTA solution, with three parallel determinations per group. The scale inhibition rate was calculated using Equation (1):

(1) $$\eta \;=\;\frac{\rho_{2}\;-\;\rho_{1}}{\rho_{0}\;-\;\rho_{1}}$$

where *ρ*_2_ is the concentration of the experimental group, *ρ*_1_ is that of the blank control group, and *ρ*_0_ is the initial concentration in raw water (all units: mg·L^−1^).

For Ca_3_(PO_4_)_2_ scale inhibition, the test solution consisted of CaCl_2_, sodium dihydrogen phosphate (NaH_2_PO_4_), and borax, at 100 mg·L^−1^ and 5 mg·L^−1^, respectively, and reacted at 80 °C for 10 h. After cooling, the absorbance of was measured at 710 nm using a spectrophotometer (three parallel tests), and the inhibition efficiency was calculated using Equation (2):

(2) $$\eta \;=\;\frac{A_{2}\;-\;A_{1}}{A_{0}\;-\;A_{1}}$$

where *A*_2_, *A*_1_, and *A*_0_ are the absorbance of in the experimental group, blank group, and initial solution, respectively. The scale inhibition rate is defined as the percentage reduction in calcium scale deposition with PASP-S compared to the blank sample, with higher values indicating superior scale inhibition performance.

### 2.4. Determination of Dispersion Performance of Iron Oxide

The dispersion performance of PASP-S on iron oxide was determined using a static method [24]. A solution with *ρ*(Ca^2+^) = 150 mg/L and *ρ*(Fe^2+^) = 10 mg/L was prepared, with pH adjusted to approximately 9. After vigorous stirring for 15 min, the mixture was heated in a 50 °C constant temperature water bath for 5 h. The absorbance of the supernatant was measured using a spectrophotometer at 420 nm, and the transmittance was calculated accordingly.

### 2.5. Corrosion Inhibition Performance Determination

#### 2.5.1. Rotating Hanging Plate Method

The corrosion inhibition performance of PASP-S was determined in accordance with the national standard *Determination of Corrosion Inhibition Performance of Water Treatment Agents—Rotating Coupon Method* (GB/T 18175—2014) [25]. The test coupons selected were 20# standard carbon steel coupons with the following chemical composition: C (0.17%), Mn (0.35%), Cr (0.25%), Ni (0.30%), Si (0.17%), and Cu (0.25%). The remainder was Fe. The coupons had dimensions of 5.0 cm × 2.5 cm × 0.2 cm and a density of 7.85 g/cm^3^.

#### 2.5.2. Electrochemical Test

Using a three-electrode system with a 3.5% NaCl solution as the corrosion medium at 25 °C, this study investigated the corrosion inhibition mechanisms of PASP-O under different concentrations using an electrochemical workstation. The working electrode was carbon steel (W.E.), the reference electrode was saturated calomel (R.E.), and the counter electrode was platinum (C.E.).

Connect the electrode and perform an open-circuit test for 1 h to obtain the stable voltage (i.e., corrosion onset potential *E_corr_*, OCP). Within the OCP ± 250 mV range, measure the galvanic polarization curve (Tafel) at a scan rate of 0.1 mV/s. The corrosion potential difference Δ*E_corr_* is calculated using Equation (3). When Δ*E_corr_* ≤ −85 mV, the inhibitor is classified as cathodic. When Δ*E_corr_* > 85 mV, it is categorized as anodic. When Δ*E_corr_* falls between these thresholds, it is defined as mixed type [13,26]. The corrosion inhibition efficiency (*η*) and surface coverage degree (θ) can be determined using Equations (4) and (5).

(3) $$∆E_{corr}=E_{corr}\;-\;E_{corr,0}$$

In the formula, *E_corr_* is the open circuit voltage with corrosion inhibitor, and *E_corr,_*_0_ is the open circuit voltage without corrosion inhibitor.

(4) $$\eta =\frac{i_{corr}^{0}-i_{corr}}{i_{corr}^{0}}\times 100\%$$

(5) $$\theta =\frac{i_{corr}^{0}-i_{corr}}{i_{corr}^{0}}$$

In the formula, *i*^0^*_corr_* and *i_corr_* are the corrosion current with and without corrosion inhibitors respectively. The corrosion current density value is obtained by extrapolation.

### 2.6. Carbon Steel Surface Analysis

The surface morphology of the carbon steel sheet was observed using a field emission scanning electron microscope (SEM, Hitachi High-Tech Corporation, Tokyo, Japan). The surface roughness of the carbon steel sheet was analyzed using a three-dimensional morphometer. The element variation of the carbon steel sheet was analyzed using an X-ray photoelectron energy spectrum (XPS), and the corrosion products of test piece were analyzed using X-ray powder diffraction.

### 2.7. Antimicrobial Performance Determination

#### 2.7.1. Filter Paper Method

The bacterial cultures stored in a −80 °C freezer were streaked on pre-prepared LB agar plates for activation. Using a sterile pipette, the activated cultures were inoculated into 100 mL LB liquid medium and incubated overnight at 37 °C and 220 rpm on a constant temperature shaker. Subsequently, 50 μL of this culture was transferred to LB liquid medium and cultured until reaching the logarithmic phase. Sterile 50 mL solid LB medium was spread on petri dishes to dry. A 5 μL culture in the logarithmic phase was mixed with 0.7% solid LB medium and then spread onto the dried plates. After drying, filter paper was placed and polymer reagent was added. The plates were inverted and incubated at 37 °C for an overnight period. Finally, the diameter of the inhibition zone was measured.

#### 2.7.2. Dilution Coating Plate Method

The bacterial strain was preserved in an −80 °C freezer and activated by streaking on LB agar plates. Using a sterile pipette, the activated strain was inoculated into 100 mL LB liquid medium in a laminar flow hood and then incubated overnight at 37 °C with a constant temperature shaker at 220 rpm. Then, 50 μL of this culture was transferred to both polymer-free and polymer-containing LB liquid media (each sample repeated three times), with continued cultivation until the logarithmic phase. After gradient dilution of the bacterial suspension with sterile water, 100 μL was spread onto solid LB plates and incubated upside down at 37 °C in a constant temperature incubator overnight. Finally, colony counts were recorded using a colony counter and averaged.

(6) $$Antibacterial\;rate\;(\eta )=\frac{A}{B}\;\times \;100\%$$

In this formula, *A* is the number of colonies in the culture medium with inhibitor, and *B* is the number of colonies in the culture medium without inhibitor.

### 2.8. Fluorescence Performance Determination

The relationship between fluorescence intensity and PASP-S concentration was investigated using a fluorescence spectrophotometer.

### 2.9. Quantum Chemical Calculations

Based on density functional theory (DFT), using the 6–31G basis set and the B3LYP functional, we investigated the relationship between the molecular structures of PASP and its modified polymers and their scale inhibition and corrosion inhibition performance. Parameters such as Δ*E* can be calculated from *E_HOMO_* and *E_LUMO_*, and the calculation method is as follows:

(7) $$\Delta E\;=\;E_{LUMO}\;-\;E_{HOMO}$$

(8) $$IP=-\;E_{HOMO}$$

(9) $$EA=-{\;E}_{LUMO}$$

(10) $$\chi \;=\frac{\;IP+EA}{2}$$

(11) $$\eta =\frac{IP-EA}{2}$$

(12) $$\Delta N=\frac{\chi_{Fe}-\chi_{inh}}{2\;(\eta_{Fe}+{\;\eta }_{inh})}$$

In the formula, *χ_Fe_* and *χ_inh_* are the absolute electronegativity of the iron atom and the inhibitor molecule, respectively; *η_Fe_* and *η_inh_* are the total hardness of the iron atom and the inhibitor molecule, respectively [27,28,29].

## 3. Results

### 3.1. The Synthesis of PASP-S

#### 3.1.1. Synthesis of PASP-E

**Figure 1:**
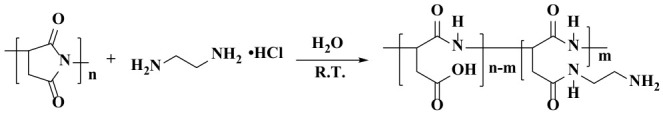
Schematic diagram of the synthesis route of PASP-E.

Precisely weigh 984 mg (10 mmol) of PSI and transfer it into a round-bottom flask. Add 10 mL of deionized water and stir to homogeneity. Transfer 7.34 mL (110 mmol) of ethylenediamine (EDA) into a beaker. Under ice bath conditions, equimolar 6 M HCl is added to neutralize part of its basicity. Upon cooling to room temperature, slowly add the resulting EDA solution into the flask. Stir magnetically at room temperature until the solution turns to a clear orange liquid (ca. 30 min). Add an equal volume of anhydrous ethanol to the reaction mixture and allow it to stand. Decant the supernatant to obtain an orange viscous liquid. This liquid is purified using dialysis membrane under alkaline conditions (M*_WCO_* = 1000) until the dialysate conductivity decreases to 2.5 μS/cm. Upon solvent removal, intermediate PASP-E (orange solid) is obtained with a yield of 78.26%. The synthetic route of PASP-E is illustrated in Figure 1.

#### 3.1.2. Synthesis of PASP-S

**Figure 2:**
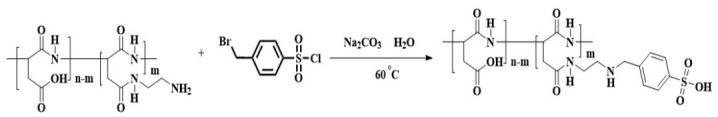
Schematic diagram of the synthesis route of PASP-S.

Precisely weigh 100 mg PASP-E and 679.2 mg (2.52 mmol) 4-bromomethylbenzenesulfonyl chloride into a 100 mL round-bottom flask. Add 10 mL deionized water, immerse the flask in a 60 °C oil bath, and stir magnetically for 24 h. During the reaction, Na_2_CO_3_ solution is added dropwise to maintain the system at weak alkalinity (pH 8–9). The Na_2_CO_3_ solution is prepared by dissolving 1.0017 g (9.45 mmol) anhydrous Na_2_CO_3_ in 5 mL deionized water. After the reaction completion, the product is purified using a dialysis membrane (M*_WCO_* = 1000) until the dialysate conductivity decreases to 2.5 μS/cm. Upon solvent removal, yellow solid PASP-S is afforded with a yield of 35.8%. The synthetic route of PASP-S is illustrated in Figure 2.

### 3.2. Structural Characterization of PASP-S

#### 3.2.1. FT-IR Spectrum

**Figure 3:**
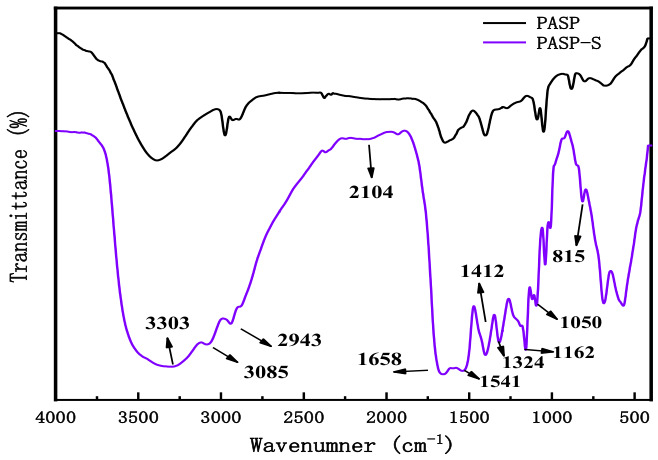
Infrared spectra of PASP and PASP-S.

The FT-IR spectra of PASP and PASP-S are presented in Figure 3. The spectral analysis of PASP-S is described as follows. The absorption peak at 3303 cm^−1^ corresponds to the stretching vibration of hydrogen-bonded hydroxyl groups (-OH). The peaks at 3085 cm^−1^ and 2943 cm^−1^ are assigned to the stretching vibrations of –CH– and –CH_2_– groups, respectively. The band at 2104 cm^−1^ belongs to the overtone absorption of benzene rings. The characteristic peak at 1658 cm^−1^ is attributed to the stretching vibration of carboxyl C=O. In addition, the peak at 1541 cm^−1^ originates from the C=O stretching vibration in amide groups, and the absorption at 1412 cm^−1^ is ascribed to the C-N stretching vibration of amide structures. Furthermore, the absorption band at 1324 cm^−1^ is assigned to the asymmetric stretching vibration of S=O bonds in sulfonic acid groups. The overlapping peaks at 1162 cm^−1^ and 1050 cm^−1^ correspond to the symmetric stretching vibration of S=O and the stretching vibration of S-O bonds, respectively [30,31]. All the above results confirm the successful the synthesis of PASP-S.

#### 3.2.2. ^1^HNMR Spectrogram

**Figure 4:**
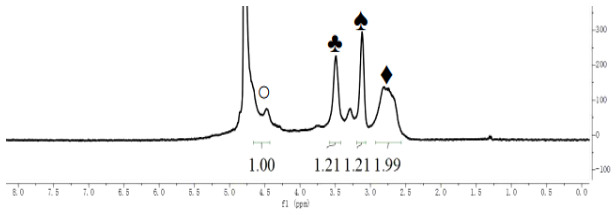
^1^H NMR spectrum of PASP-E.

The ^1^H NMR spectrum of PASP-E in deuterated water is shown in Figure 4. The peaks at δ = 3.0–3.2 ppm (♠) and δ = 3.4–3.6 ppm (♣) correspond to the –CH_2_–CH_2_– groups in EDA, while the peaks at δ = 2.6–3.0 ppm (♦) and δ = 4.4–4.7 ppm (○) are attributed to the –CH_2_– and –CH– groups in PASP [32]. These results confirm the successful preparation of the intermediate PASP-E. According to Equation (13) and the NMR integral areas, the grafting ratio of ethylenediamine is calculated to be approximately 60% [33].

(13) $$Degree\;of\;substitution=\frac{Area(H\;in\;ethylenediamine\;group)/2}{Area(H\;in\;PASP\;backbone)/2}$$

**Figure 5:**
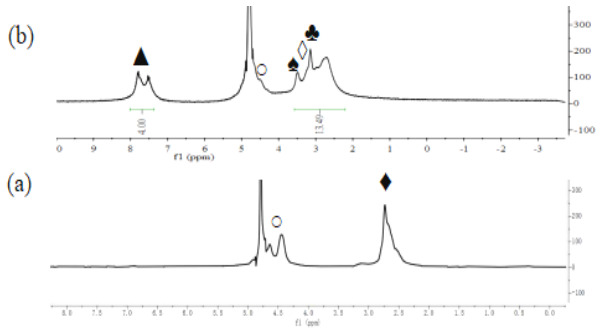
(**a**) PASP ^1^HNMR spectrogram, (**b**) PASP-S ^1^HNMR spectrogram.

The ^1^H NMR spectra of PASP and PASP-S (solvent: D_2_O) are presented in Figure 5. The characteristic peaks at δ = 2.25–2.8 ppm (♦) and δ = 4.3–4.4 ppm (○) are attributed to the –CH_2_– and –CH– groups in the PASP backbone, respectively. The peaks at δ = 3.0–3.15 ppm (♣) and δ = 3.25–3.5 ppm (♠) correspond to the –CH_2_–CH_2_– groups in EDA, while the peak at δ = 3.15–3.25 ppm (◊) is assigned to the –CH_2_– group in 4-(bromomethyl)phenylacetic acid. Additionally, the characteristic absorption peaks of the benzene ring in the aromatic region appear at δ = 7.2–7.85 ppm (▲). Collectively, the results of FT-IR and ^1^H NMR confirm the successful synthesis of PASP-S. According to Equation (14) and the NMR integral area, the grafting rate of PASP-S was calculated to be approximately 38.5% [33].

(14) $$Sulfonic\;acid\;content=\frac{Area(H\;in\;Benzene\;ring)/4}{Area(H\;in\;PASP\;backbone)/2}$$

#### 3.2.3. GPC Analysis

**Figure 6:**
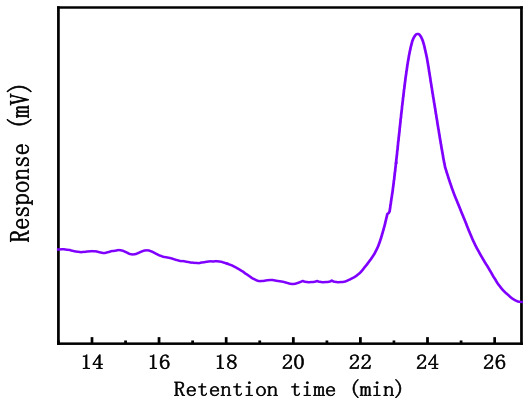
PASP-S gel permeation chromatography.

The molecular weight distribution of PASP-S was determined using gel chromatography. The results are shown in Figure 6. The number-average molecular weight (M_n_) is 1783, the weight-average molecular weight (M_w_) was 2162, and the dispersion coefficient PDI was 1.21. These results indicate that the average molecular weight distribution of PASP-S was narrow and the dispersion was good.

### 3.3. The Scale Inhibition Performance of PASP-S

#### 3.3.1. Analysis of CaCO_3_ Scale Inhibition Performance

**Figure 7:**
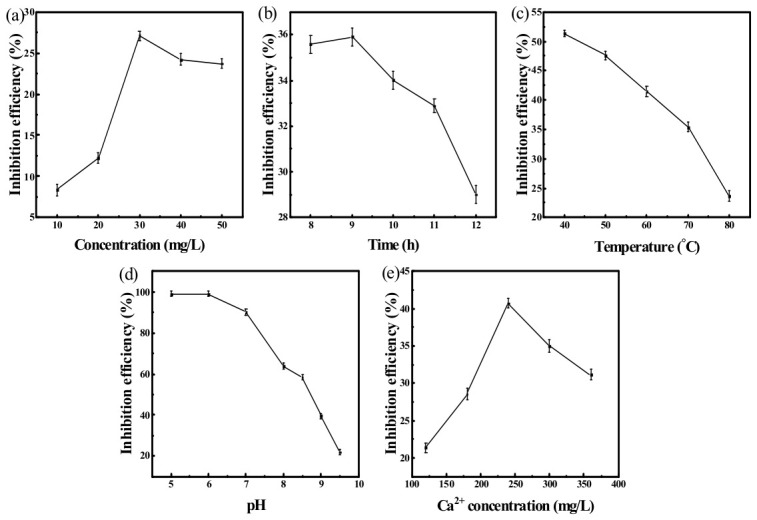
Effect of different factors on the CaCO_3_ scale-inhibition effect of PASP-S: (**a**) scale inhibitor concentration, (**b**) heating duration, (**c**) test temperature, (**d**) solution pH value, (**e**) solution Ca^2+^ potency.

Figure 7a shows the effect of PASP-S concentration on the scale inhibition performance against CaCO_3_ scale. With the increase in PASP-S concentration, the inhibition efficiency of CaCO_3_ scale first rises and then presents a threshold effect, while the scale inhibition efficiency decreases slightly after the concentration exceeds 30 mg/L. The scale inhibition rate is 12.23% at 20 mg/L and reaches 27.10% at 30 mg/L. At low concentrations, the functional groups on PASP-S molecules can chelate with Ca^2+^ and adsorb onto the surface of CaCO_3_ microcrystals, thereby interfering with the regular crystal growth. The increase in dosage provides more active sites and further improves the scale inhibition effect. Accordingly, 30 mg/L was selected as the characteristic concentration for further investigation on its scale inhibition stability.

Figure 7b shows that the inhibition efficiency of PASP-S against CaCO_3_ scale gradually decreases with the extension of heating time. The scale inhibition rate is 35.59% after heating for 8 h and drops to 32.89% when the heating time is prolonged to 11 h. The long-term thermal effect weakens the molecular structural stability of PASP-S, reduces its chelating capacity for Ca^2+^ as well as its regulatory effect on crystal growth, and thereby fails to effectively inhibit the nucleation, precipitation, and deposition of CaCO_3_ crystals.

As shown in Figure 7c, the scale inhibition performance of PASP-S against CaCO_3_ gradually declines with the increase in temperature. The scale inhibition rate is 51.35% at 40 °C and decreases to 23.62% as the temperature rises to 80 °C. Elevated temperature weakens the chelating ability of functional groups in PASP-S and their adsorption capacity on crystal planes. Meanwhile, it accelerates the nucleation and growth rate of crystals and thus promotes the rapid formation of CaCO_3_ scale.

As shown in Figure 7d, the inhibition efficiency of PASP-S against CaCO_3_ scale gradually decreases with increasing solution pH. An acidic environment can inhibit the precipitation of calcium carbonate. Under weakly alkaline conditions, the functional groups of PASP-S undergo deprotonation, which weakens their chelating and adsorption capacities. In alkaline systems, carbonate ions are more readily generated, accelerating the nucleation and growth of CaCO_3_ crystals and consequently leading to the attenuation of scale inhibition performance.

As shown in Figure 7e, with the increase in Ca^2+^ concentration, the scale inhibition rate of PASP-S against CaCO_3_ scale first rises and then declines. When the Ca^2+^ concentration reaches 240 mg/L, the scale inhibition rate remains at 40.72%. This indicates that PASP-S can still exert favorable chelating and crystal growth inhibition effects under moderately high Ca^2+^ concentrations and that it possesses certain application potential for water quality with high concentration ratios.

#### 3.3.2. Analysis of CaSO_4_ Scale Inhibition Performance

**Figure 8:**
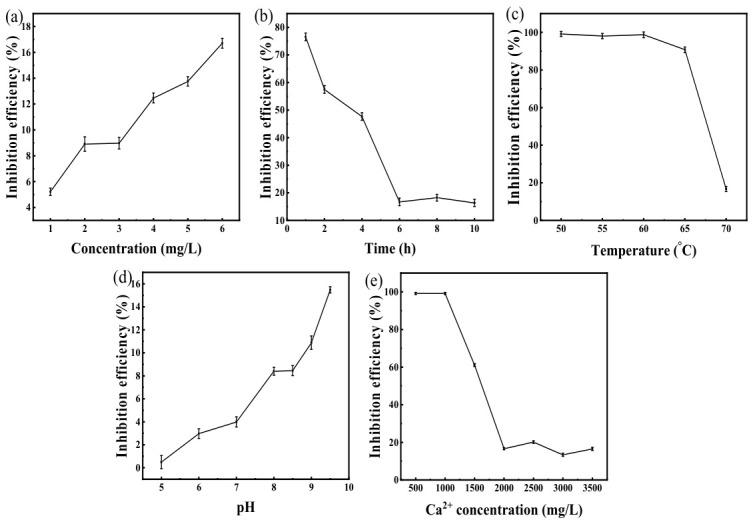
Effect of different factors on the CaSO_4_ scale-inhibition effect of PASP-S: (**a**) scale inhibitor concentration, (**b**) heating duration, (**c**) solution temperature, (**d**) solution pH value, (**e**) solution Ca^2+^ potency.

Figure 8 systematically explores the inhibition performance of PASP-S against CaSO_4_ scale. Figure 8a indicates that the inhibition efficiency of PASP-S on CaSO_4_ scale increases gradually with the rise in concentration. The scale inhibition rate is 8.97% at 3 mg/L and rises to 16.70% at 6 mg/L. As the dosage of the inhibitor increases, the number of effective functional groups in the system grows, which enhances the chelating effect on Ca^2+^ and the adsorption capacity on the surface of CaSO_4_ microcrystals. Therefore, 6 mg/L was selected as the characteristic concentration for subsequent stability investigation.

It can be observed from Figure 8b that the CaSO_4_ scale inhibition efficiency of PASP-S decreases continuously with the extension of heating time. The scale inhibition rate is 76.56% after heating for 1 h, drops to 47.65% at 4 h, and only remains 16.70% at 6 h. Long-term thermal action destroys the molecular structural stability of PASP-S, weakens its ability to chelate Ca^2+^ and interfere with the regular growth of gypsum crystals, and thus fails to effectively inhibit crystal nucleation, precipitation, and deposition.

In Figure 8c, temperature presents an initially stable influence on the scale inhibition effect followed by a sharp decline. The scale inhibition rate remains above 90% in the range of 50–65 °C and drops sharply to 16.70% when the temperature rises to 70 °C. At an appropriate temperature, PASP-S can stably exert chelation and crystal plane adsorption effects. Excessively high temperature causes partial instability of the molecular structure of PASP-S and reduces the activity of functional groups, thereby significantly weakening its inhibitory effect on the growth of CaSO_4_ crystals.

Figure 8d shows that pH exerts a remarkable influence on the CaSO_4_ scale inhibition performance of PASP-S. Under acidic conditions, inhibitor molecules are protonated, resulting in fewer effective complexation and adsorption sites and relatively low scale inhibition efficiency. In weakly alkaline environments, the degree of molecular deprotonation increases, accompanied by more active anionic functional groups. This strengthens the chelating ability toward Ca^2+^ and the regulation effect on crystal growth, so the scale inhibition performance is greatly improved.

Figure 8e reveals that the CaSO_4_ scale inhibition efficiency of PASP-S decreases gradually with the increase in Ca^2+^ concentration. The scale inhibition rate reaches 100% at a Ca^2+^ concentrations of 500–1000 mg/L, decreases to 61.10% at 1500 mg/L, and falls to only 16.62% at 2000 mg/L. A high Ca^2+^ concentration makes the ionic product of the solution exceed the solubility product of CaSO_4_, which greatly enhances the tendency of nucleation and crystallization. Meanwhile, excessive Ca^2+^ consumes the active functional groups of PASP-S, weakens its chelation and crystal growth inhibition effects, and leads to a continuous decline in scale inhibition performance.

#### 3.3.3. Analysis of Ca_3_(PO_4_)_2_ Scale Inhibition Performance

**Figure 9:**
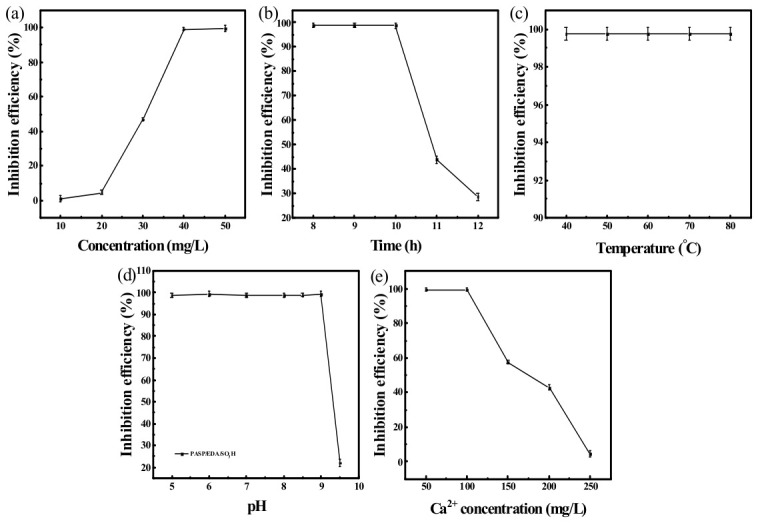
Effect of different factors on the Ca_3_(PO_4_)_2_ scale-inhibition effect of PASP-S: (**a**) scale inhibitor concentration, (**b**) heating duration, (**c**) solution temperature, (**d**) solution pH value, (**e**) solution Ca^2+^ potency.

Figure 9a illustrates the effect of PASP-S concentration on Ca_3_(PO_4_)_2_ scale inhibition. The efficiency rises with increasing concentration, reaching 46.91% at 30 mg/L and nearly 100% at 50 mg/L. Mechanistically, PASP-S effectively disperses Ca^2+^ and PO_4_^3−^ in the solution, reducing their collision probability and inhibiting Ca_3_(PO_4_)_2_ scale deposition. Consequently, 50 mg/L was selected to investigate the stability of PASP-S’s Ca_3_(PO_4_)_2_ scale inhibition performance.

Figure 9b depicts the effect of heating duration on the Ca_3_(PO_4_)_2_ scale inhibition efficiency of PASP-S, which gradually decreases with prolonged heating. The inhibition rate remains nearly 100% within the first 10 h of heating, drops sharply to 43.84% at 11 h, and further declines to 28.74% at 12 h. The experimental results indicate that PASP-S exhibits a certain degree of thermal stability over short periods. However, as the heating duration increases, its thermal stability and ability to disperse Ca^2+^ and PO_4_^3−^ gradually weaken, ultimately accelerating the formation of Ca_3_(PO_4_)_2_ scale.

Figure 9c shows the effect of solution temperature on the Ca_3_(PO_4_)_2_ scale inhibition efficiency of PASP-S. The inhibition rate remained basically stable with the increase in temperature, and PASP-S inhibited Ca_3_(PO_4_)_2_. The scale deposition shows excellent temperature tolerance, and it can effectively disperse the scale-forming ions in the solution within the test temperature range, reduce the probability of collision between them, and improve PASP-S resistance to Ca_3_(PO_4_)_2_ scale performance.

The inhibition efficiency of PASP-S against Ca_3_(PO_4_)_2_ scale in test solutions with different pH values is shown in Figure 9d. When PASP-S is used for Ca_3_(PO_4_)_2_, the inhibition efficiency decreases with the increase in the pH value of the solution. When pH < 7, H^+^ ions take part in the Ca_3_(PO_4_)_2_ reaction and generate H_3_PO_4_ and CaCl_2_, while PO_4_^3−^ remains in the free state in solution. When pH = 7–9, H^+^ takes part in the PO_4_^3−^ insufficient reaction, resulting in soluble calcium dihydrogen phosphate. When pH > 9, OH^−^ reacts with Ca^2+^ to form Ca(OH)_2_ and guide Ca_3_(PO_4_)_2_ deposition, resulting in a decrease in the scale inhibition rate.

Figure 9e illustrates the effect of Ca^2+^ concentration on the scale inhibition performance. The scale inhibition rate gradually decreases with the increase in Ca^2+^ concentration. The scale inhibition rate remains close to 100% at Ca^2+^ concentrations ranging from 50 to 100 mg/L and declines to 57.5% when the concentration rises to 150 mg/L. As the Ca^2+^ concentration increases, the ionic product of the solution exceeds the solubility product of Ca_3_(PO_4_)_2_, which enhances the tendency of nucleation and crystallization. Excess Ca^2+^ consumes the active functional groups of PASP-S and weakens its chelating capacity as well as its ability to restrain grain agglomeration and growth, thereby resulting in a continuous decline in scale inhibition efficiency.

### 3.4. Scale Analysis

#### 3.4.1. XRD Test

**Figure 10:**
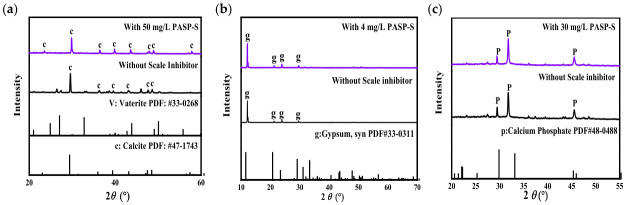
XRD diagram of calcium scale crystals: (**a**) CaCO_3_ scale, (**b**) CaSO_4_ scale, (**c**) Ca_3_(PO_4_)_2_ scale.

Figure 10a shows the XRD patterns of CaCO_3_ scale crystals formed without and with the addition of 30 mg/L PASP-S. By analyzing the main characteristic strong diffraction peaks and comparing them with the standard PDF card, it can be seen that the CaCO_3_ scale without PASP-S is mainly calcite in crystal form. After the addition of PASP-S, the characteristic diffraction peaks of calcite are still retained, and no new crystalline phase diffraction peaks or obvious peak position shifts are observed. The results indicate that PASP-S does not change the crystal type of the CaCO_3_ scale.

Figure 10b presents the XRD patterns of CaSO_4_ scale crystals formed in the absence and presence of 4 mg/L PASP-S. Analysis of the main characteristic peaks shows that without PASP-S, distinct diffraction peaks appear at 2θ = 11.58°, 20.72°, 23.39°, and 28.10°, which correspond to CaSO_4_·2H_2_O (gypsum) according to a standard PDF card comparison. After adding 4 mg/L PASP-S, the crystal form of CaSO_4_ scale remains CaSO_4_·2H_2_O with no alteration in crystal type, demonstrating that PASP-S cannot change the crystal structure of gypsum scale.

Figure 10c displays the XRD patterns of Ca_3_(PO_4_)_2_ scale crystals without and with 30 mg/L PASP-S. According to the analysis of main characteristic peaks and standard card comparison, obvious diffraction peaks appear at 2θ = 29.85°, 33.15°, and 45.35° for Ca_3_(PO_4_)_2_ scale regardless of PASP-S addition, which are assigned to calcium phosphate. No new diffraction peaks or peak shifts emerge after PASP-S addition, confirming that PASP-S does not alter the crystal form of Ca_3_(PO_4_)_2_ scale.

#### 3.4.2. SEM Analysis

**Figure 11:**
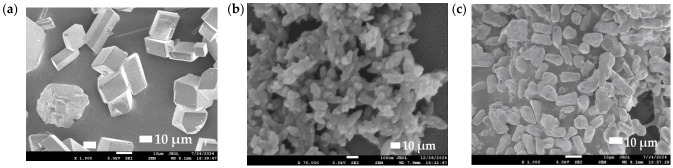
CaCO_3_ SEM images: (**a**) no scale inhibitor, (**b**) 30 mg/L PASP-S, (**c**) 50 mg/L PASP-S.

CaCO_3_ scale: Figure 11a shows the CaCO_3_ scale formed without the addition of PASP-S. The crystal morphology presents typical calcite features, which is consistent with the XRD results. Figure 11b displays the CaCO_3_ scale formed with 30 mg/L PASP-S. The crystal surface is obviously destroyed; the original hexahedral structure and compact surface morphology disappear, and the crystals transform into scattered and irregular particles. As shown in Figure 11c, when the PASP-S concentration is increased to 50 mg/L, the surface damage degree of CaCO_3_ scale crystals becomes more severe. PASP-S can adsorb onto the surface of calcium scale crystals and destroy their morphological structure. Moreover, the degree of surface damage to the calcium scale increases with the rise in PASP-S concentration.

**Figure 12:**
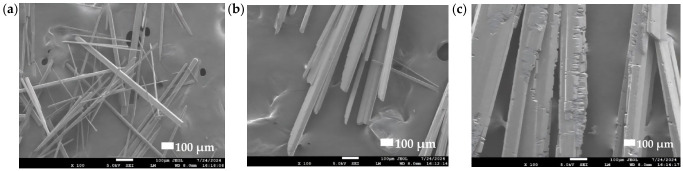
CaSO_4_ scale SEM diagram: (**a**) no scale inhibitor, (**b**) 2 mg/L PASP-S, and (**c**) 4 mg/L PASP-S.

CaSO_4_ Scale: Figure 12a shows the CaSO_4_ scale formed without PASP-S. The scale crystals, with a smooth and flat surface, appear as needle-like crystals. After adding 2 mg/L PASP-S, as illustrated in Figure 12b, the CaSO_4_ scale crystals exhibit an obvious agglomeration phenomenon, and the crystal surface is damaged and roughened. After adding 4 mg/L PASP-S, as illustrated in Figure 12c, the damage degree of the CaSO_4_ scale crystal surface is aggravated, and sawtooth edges appear. PASP-S can adsorb on the surface of scale crystals, occupy their growth sites, and inhibit the growth of crystal planes to a certain extent.

**Figure 13:**
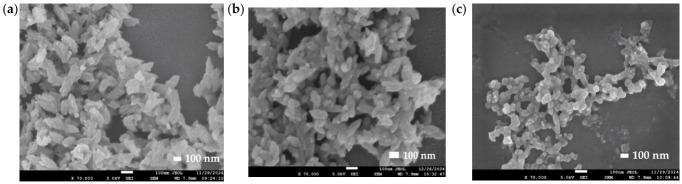
Ca_3_(PO_4_)_2_ scale SEM diagram: (**a**) no scale inhibitor, (**b**) 10 mg/L PASP-S, and (**c**) 30 mg/L PASP-S.

Ca_3_(PO_4_)_2_ Scale: Figure 13a shows the Ca_3_(PO_4_)_2_ scale formed without PASP-S; its morphology is mainly small spindle-shaped nanoparticles. Figure 13b shows the morphology of the Ca_3_(PO_4_)_2_ scale formed after adding 10 mg/L PASP-S. The Ca_3_(PO_4_)_2_ scale still exists in the form of spindle-shaped particles. However, the particle size is reduced, and the particles are more dispersed. The morphology of the Ca_3_(PO_4_)_2_ scale crystals formed after adding 30 mg/L PASP-S is shown in Figure 13c. The size of the Ca_3_(PO_4_)_2_ scale crystals is further reduced, and the particles are more dispersed. PASP-S interacts with scale-forming ions in the dispersed aqueous solution and reduces the collision between them, thus inhibiting scale formation.

**Figure 14:**
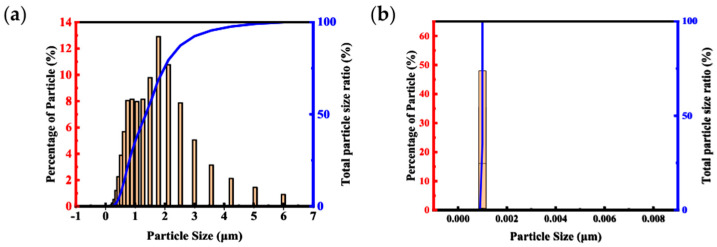
Ca_3_(PO_4_)_2_ dynamic light scattering (DLS) particle size distribution of scale: (**a**) without antiscalant, (**b**) with 30 mg/L PASP-S.

Figure 14 shows the particle size distribution of Ca_3_(PO_4_)_2_ scale particles in the test solution measured using the DLS method. Figure 14a presents the particle size distribution of Ca_3_(PO_4_)_2_ scale particles without PASP-S, indicating that the particle size of the calcium scale is relatively wide and mainly concentrated in the range of 0–3 μm. In Figure 14b, after adding 30 mg/L PASP-S, the size of Ca_3_(PO_4_)_2_ scale particles decreases significantly, with the particle size mainly distributed around 0.001 μm, which is consistent with the particle size variation trend observed using SEM.

### 3.5. Dispersion of Iron Oxide in PASP-S

**Figure 15:**
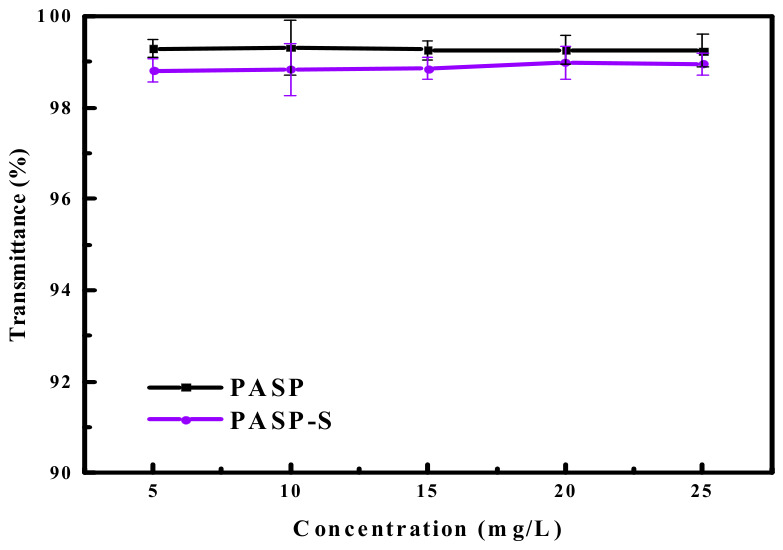
Effect of PASP and PASP-S concentration on solution transmittance.

Figure 15 shows the influence of PASP-S concentration on the dispersion performance of iron oxide. PASP does not have the ability to disperse iron oxide.

After adding different concentrations of PASP-S, the transmittance of the test solution decreased slightly with the increase in PASP-S concentration. When the concentration of PASP-S was 15 mg/L, the transmittance of the solution was 98.85%, indicating that PASP-S does not have the ability to disperse iron oxide.

### 3.6. Corrosion Inhibition Performance of PASP-S

#### 3.6.1. Rotating Hanging Plate Method

**Figure 16:**
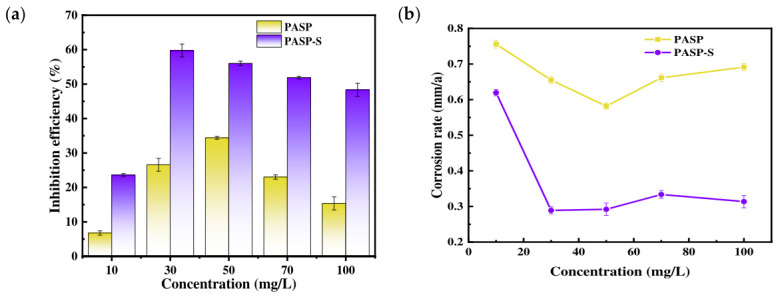
Corrosion inhibition performance of PASP and PASP-S: (**a**) corrosion inhibition efficiency, (**b**) corrosion rate.

The corrosion inhibition performances of PASP and PASP-S at 45 °C were determined using the rotating coupon method, and the results are illustrated in Figure 16. Figure 16a demonstrates the effects of PASP and PASP-S concentrations on corrosion inhibition efficiency. The addition of PASP-S significantly enhanced the inhibition efficiency compared to PASP, showing an initial increase followed by a gradual decline with higher concentrations. At 30 mg/L, the inhibition rate reached its maximum value of 59.77%, representing a 33.19% improvement over PASP. The adsorption of PASP-S on the test specimen surface blocked contact between the corrosive medium and the specimen surface. When the concentration was increased to 50 mg/L, the inhibition rate began to decline gradually, with desorption occurring on the carbon steel test specimen surface. However, the overall inhibition efficiency remained above 50%, still outperforming PASP. Figure 16b illustrates the effects of PASP and PASP-S concentrations on corrosion rate. The addition of PASP-S significantly reduced the corrosion rate, achieving the lowest rate of 0.2889 mm/year at 30 mg/L, which is 0.3661 mm/year lower than PASP at the same concentration.

#### 3.6.2. Carbon Steel Test Piece Analysis

Test Piece Morphology Analysis: The surface morphology of carbon steel test pieces before and after corrosion was analyzed using SEM and a three-dimensional contour morphology instrument, and the results are shown in Figure 15.

**Figure 17:**
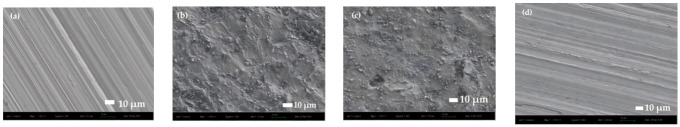
SEM images of specimens: (**a**) polished, (**b**) without inhibitors, (**c**) with the addition of 50 mg/L PASP, (**d**) with the addition of 50 mg/L PASP-S.

Figure 17a shows the polished surface morphology of carbon steel specimens, which present a smooth surface with regular metallic striations. Figure 17b displays an SEM image of carbon steel specimens immersed in inhibitor-free corrosion solution, revealing extremely rough surfaces that have lost their original striations, with numerous loose corrosion products adhering to the metal surface. Figure 17c presents SEM images of carbon steel specimens immersed in a 50 mg/L PASP corrosion solution. While the surface roughness is reduced compared to the control group, loose corrosion products remain present along with localized pitting. Figure 17d shows SEM images of specimens immersed in a 50 mg/L PASP-S corrosion medium. The specimens exhibit significantly smoother surfaces with preserved original striations, markedly reduced corrosion products, and substantially diminished pitting. The PASP-S formulation demonstrates enhanced corrosion inhibition performance, providing effective protection for carbon steel specimens.

**Figure 18:**
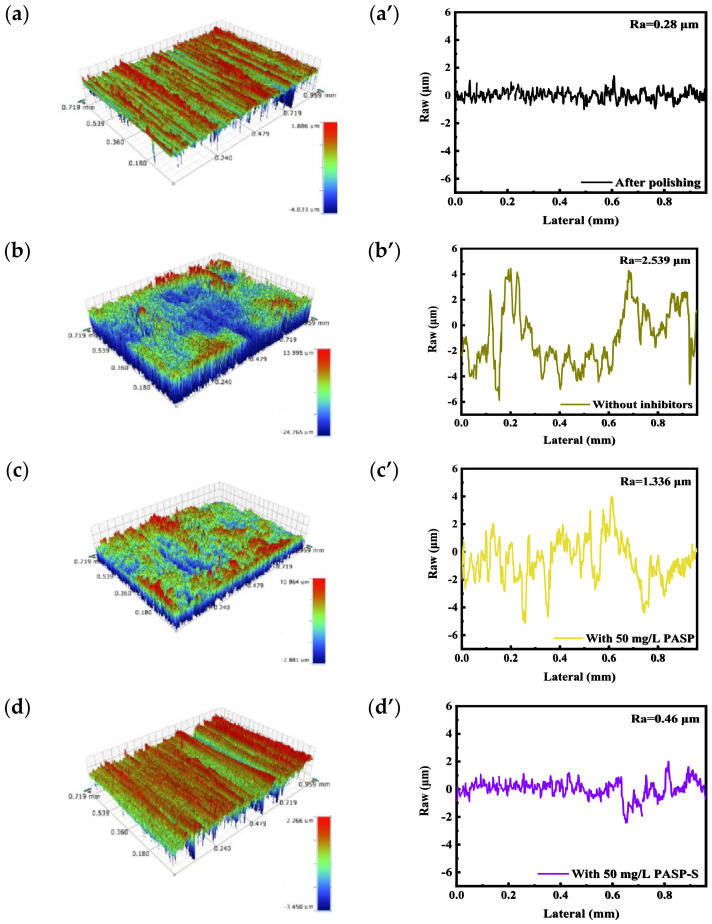
Three–dimensional morphology of specimens and two-dimensional cross–sectional curves: (**a**,**a’**) polished, (**b**,**b’**) without inhibitor, (**c**,**c’**) with the addition of 50 mg/L PASP, (**d**,**d’**) with the addition of 50 mg/L PASP-S.

Figure 18 displays the 3D profile morphology and 2D cross-sectional curves of the carbon steel coupons, which intuitively reflect the groove depth and surface roughness of the coupons. Figure 18a presents the surface profile of the polished coupon, featuring uniform roughness, intact metallic texture, and an average groove depth of 0.28 μm (Figure 18a’). Figure 18b shows the surface profile of the coupon immersed in the inhibitor-free corrosive medium; the coupon surface suffered severe corrosion, losing its original metallic texture and becoming rough and non-uniform, with an average groove depth of 2.539 μm (Figure 18b’). The surface profile of the coupon soaked in the corrosive medium containing 50 mg/L PASP is illustrated in Figure 18c, where the surface roughness was reduced and the average groove depth reached 1.336 μm (Figure 18c’). When the concentration of PASP-S in the corrosive medium was 50 mg/L, the 3D profile of the coupon is shown in Figure 18d. The coupon surface exhibited a regular metallic texture and smoothness, which was similar to that of the pre-corrosion coupon, with an average groove depth of 0.46 μm (Figure 18d’). These results indicate that PASP-S possesses excellent corrosion inhibition performance, as it can form a protective film on the carbon steel surface to block the direct contact between the coupon surface and the corrosive medium, thereby preventing the coupon from corrosion.

**Figure 19:**
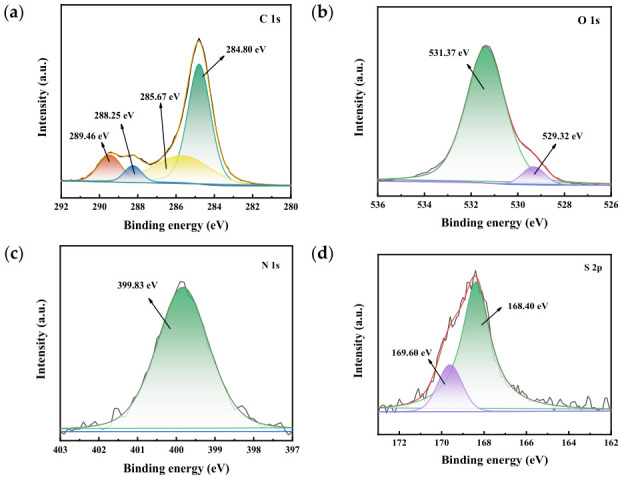
XPS energy spectrum of carbon steel test pieces immersed in corrosion medium containing 50 mg/L PASP-S for 72 h: (**a**) C 1s, (**b**) O 1s, (**c**) N 1s, (**d**) S 2p.

XPS Analysis: When the PASP-S concentration was 50 mg/L, surface analysis of the uncorroded areas of test specimens after corrosion experiments was conducted, as shown in Figure 19. Deconvolution curves of C, O, N, and S elements were obtained via analysis. Specifically, the deconvolution curve of C 1s exhibited four characteristic peaks. The peak at 284.80 eV could be assigned to the C–H, C–C, and C=C bonds in the PASP-S molecule. The peak at 285.67 eV corresponded to the C–O or C–N bond, and the peaks at 288.25 eV and 289.46 eV were attributed to the O–C=O and N–C=O bonds, respectively [34,35]. The deconvolution curve of O 1s showed two peaks at 529.32 eV and 531.37 eV, which were assigned to the iron oxides on the coupon surface and the C=O bonds in the PASP-S molecule, respectively [36]. A single peak was observed in the deconvolution curve of N 1s at 399.83 eV, corresponding to the C–N–C bond [37]. For the S 2p deconvolution curve, two peaks were detected at 168.40 eV and 169.60 eV, which were ascribed to S 2p3/2 and S 2p1/2, respectively [38]. Surface analysis of the coupons confirmed the presence of PASP-S molecules on the coupon surface, which adsorbed onto the surface to form a protective film and thereby prevent the corrosive medium from eroding the coupons.

#### 3.6.3. Electrochemical Analysis

**Figure 20:**
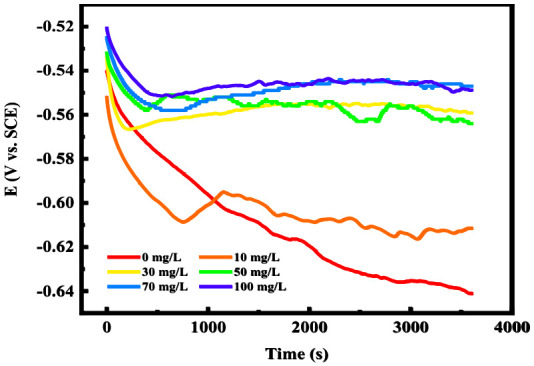
Open–circuit potential versus time curves of carbon steel electrodes in 3.5% NaCl solutions containing different concentrations of PASP-S.

Open Circuit Potential (OCP): The time-dependent curve of open circuit potential in a 3.5% NaCl corrosion medium containing varying concentrations of PASP-S is shown in Figure 20 with relevant parameters listed in Table 1. The corrosion voltage increases with higher PASP-S concentrations. At low concentrations, PASP-S functions as a mixed-type corrosion inhibitor that simultaneously suppresses both the anodic dissolution of carbon steel and the cathodic hydrogen evolution reaction. At high concentrations, it acts as an anodic-type corrosion inhibitor, where PASP-S adsorbs on the electrode surface to form a protective film, enhancing charge transfer resistance and thereby inhibiting carbon steel dissolution.

**Figure 21:**
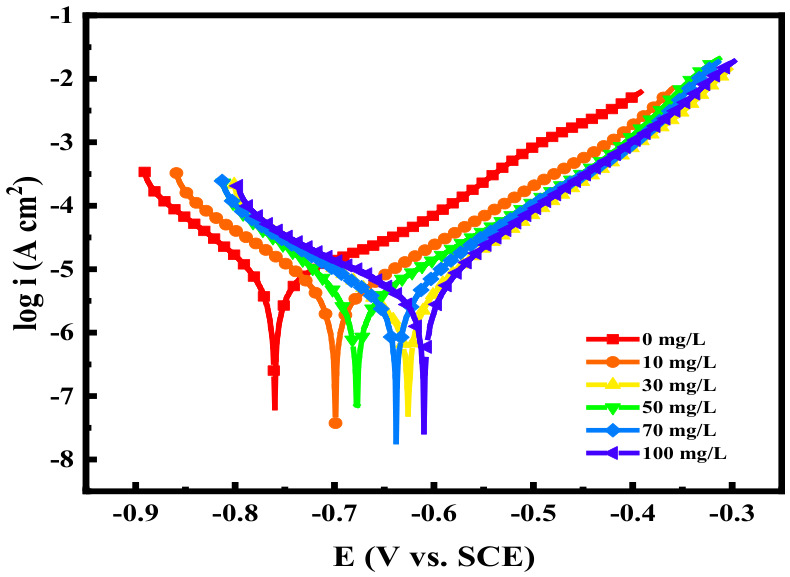
Tafel curves of carbon steel electrodes in 3.5% NaCl solutions containing different concentrations of PASP-S.

Potentiostatic Polarization Curve (Tafel): After establishing a stable open-circuit potential, the carbon steel electrode underwent further potentiostatic polarization testing as shown in Figure 21. When the corrosion medium contained different concentrations of PASP-S, both the anodic and cathodic regions of the Tafel curve exhibited current density shifts toward lower values. Significant changes occurred simultaneously in both zones, indicating that the adsorption of PASP-S on the electrode surface formed a protective layer. This enhanced charge transfer resistance in the solution, effectively suppressing the dissolution reaction of carbon steel in corrosive media. Additionally, at low concentrations, the addition of PASP-S partially inhibited hydrogen evolution at the cathode, further mitigating corrosion. Based on the Tafel curves, relevant electrochemical parameters were extrapolated (Table 2). The data reveal that as the inhibitor concentration increased, the corrosion current gradually decreased, while coverage and corrosion inhibition efficiency progressively improved.

### 3.7. Antibacterial Properties of PASP-S

Given that industrial circulating water systems operate as semi-open systems with direct water-body contact, the presence of inorganic salts and organic matter in the water creates favorable conditions for microbial growth, leading to bacterial proliferation. For example, metabolites secreted by bacteria such as *Escherichia coli* can induce the formation of microbial slime, thereby aggravating pipeline corrosion. The circulating water primarily contains *Escherichia coli* (*E. coli*), *Staphylococcus aureus* (*S. aureus*), and *Bacillus cereus* (*B. subtilis*). This study employed the filter paper method and dilution spread plate method to evaluate the antibacterial efficacy of PASP and PASP-S against these three microorganisms.

#### 3.7.1. Filter Paper Method

**Figure 22:**
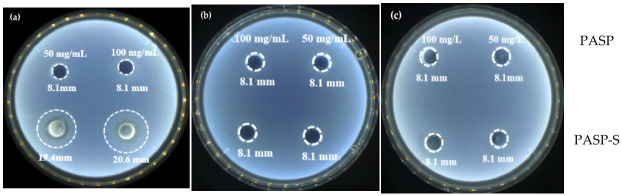
Effect diagram of the inhibition of PASP and PASP-S on different bacterial species: (**a**) *Escherichia coli*, (**b**) *Staphylococcus aureus*, (**c**) *Bacillus cereus*.

The effects of PASP and PASP-S concentrations on the inhibition zone diameters of *Escherichia coli*, *Staphylococcus aureus*, and *Bacillus cereus* are presented in Figure 22. As shown in Figure 22a, both PASP and PASP-S exerted relatively weak inhibitory activity against *E. coli* colonies. When the culture medium contained 50 mg/mL of PASP-S, the inhibition zone diameter for *E. coli* reached 19.4 mm, which was 11.3 mm larger than that of PASP at the same concentration. With a further increase in concentration to 100 mg/mL, the inhibition zone expanded to 20.6 mm, representing a 12.5 mm increase compared to PASP. These results confirm that PASP-S possesses superior inhibitory capacity against *E. coli*, outperforming PASP in both the strength and scope of suppression.

The inhibition zone images of PASP and PASP-S against *Staphylococcus aureus* are displayed in Figure 22b. The results indicate that PASP exhibited poor inhibitory efficacy against *Staphylococcus aureus* colonies. When different concentrations of PASP-S were added to the culture medium, the inhibition zone diameter remained constant at 8.1 mm, suggesting that PASP-S exerts a strongly specific bacteriostatic effect on *Staphylococcus aureus*.

Figure 22c shows the inhibition zone images of *Bacillus cereus* when 50 mg/mL and 100 mg/mL of PASP and PASP-S were added to the culture medium, separately. Notably, PASP-S exhibited no inhibitory effect on *Bacillus cereus*.

Based on the inhibitory effects of PASP-S on the three bacterial strains, PASP-S demonstrates high antibacterial specificity. It exerts strong inhibitory activity against *E. coli*, weak inhibitory activity against *Staphylococcus aureus*, and no effect on *Bacillus cereus*. This specificity enables it to regulate bacterial communities in circulating water to a certain extent.

#### 3.7.2. Filter Paper Method

**Figure 23:**
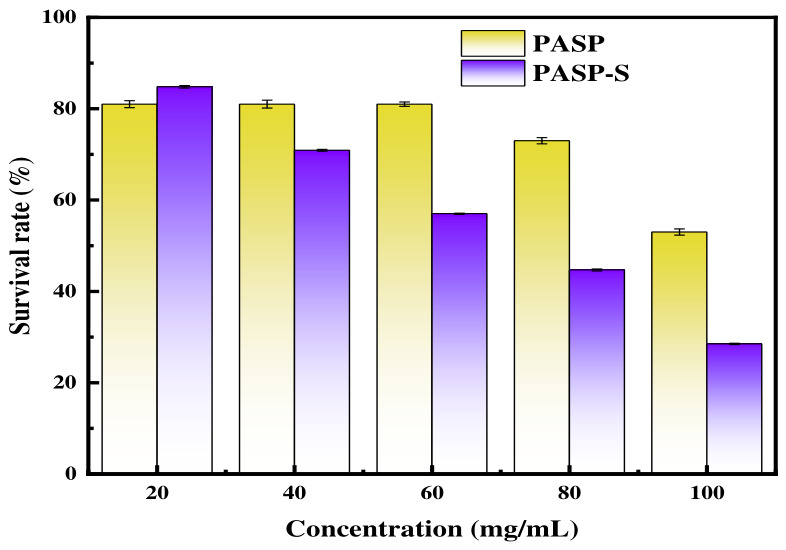
Influence of PASP and PASP-S concentration on the survival rate of *E. coli*.

The effects of PASP and PASP-S on *Escherichia coli* (*E. coli*) survival were evaluated using the dilution plate method (Figure 23). As shown in the figure, the survival rate of *E. coli* gradually decreased with increasing concentrations of PASP-S. At low concentrations, PASP had a minimal impact on *E. coli* survival, while PASP-S showed a significant effect. When the concentration of PASP-S reached 60 mg/mL, the survival rate of *E. coli* was 57.00%, representing a decrease of 24.00% compared to PASP. At 100 mg/mL, the survival rate dropped to 28.50%, showing a reduction of 24.50% from PASP.

Based on the above, it can be seen that compared with unmodified PASP, the modified product PASP-S exhibits more excellent and specific antibacterial properties. In the antibacterial experiments against industrial circulating water, PASP-S demonstrated a significantly enhanced inhibitory effect on *Escherichia coli*. The diameter of the inhibition zone was clearly larger than that of PASP at the same concentration, and the inhibitory effect was further strengthened with the increase in concentration, reflecting good antibacterial activity and concentration dependence. Although PASP-S only showed an antibacterial effect against *Staphylococcus aureus* comparable to that of PASP and had no obvious inhibitory effect on *Bacillus cereus*, such strong selective inhibition against the Gram-negative bacterium *Escherichia coli* precisely endows it with a high degree of antibacterial specificity. In the industrial circulating water system, this specific antibacterial characteristic not only precisely controls the dominant harmful bacteria groups but also reduces the broad-spectrum interference to the environmental microbial community, featuring both high efficiency and targeting. This fully reflects the unique advantages and application potential of PASP-S as a circulating water treatment agent in antibacterial regulation.

### 3.8. Fluorescence Properties

**Figure 24:**
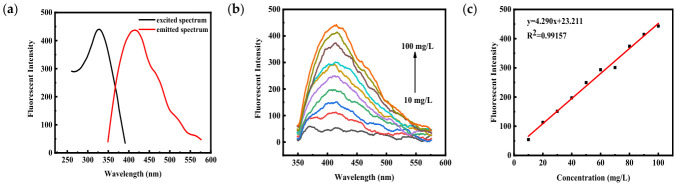
Fluorescence spectrum of PASP-S: (**a**) excitation spectrum and emission spectrum, (**b**) effect of concentration on fluorescence intensity, (**c**) linear fitting curve of concentration and fluorescence intensity.

Figure 24a depicts the excitation and emission spectra of PASP-S. The compound displays a maximum excitation wavelength of 325 nm and a peak emission wavelength of 410 nm, with both spectra exhibiting remarkable mirror symmetry. Notably, the emission wavelength is significantly longer than the excitation wavelength, a phenomenon attributed to the Stokes shift occurring during electron transitions.

Figure 24b illustrates the variation trend of fluorescence intensity of PASP-S with concentration ranging from 10 mg/L to 100 mg/L at an interval of 10 mg/L. It can be observed that the fluorescence intensity of PASP-S increases with the rising concentration, and no obvious fluorescence quenching occurs, indicating that PASP-S possesses favorable fluorescence stability.

Within the tested concentration range of 10–100 mg/L, a strong linear correlation exists between fluorescence intensity and inhibitor concentration, as indicated by a high correlation coefficient (R^2^ = 0.9916), endowing it with potential for real-time monitoring of scale and corrosion inhibitor concentrations in industrial circulating water.

### 3.9. Analysis of Antiscaling and Corrosion Inhibition Mechanism

#### 3.9.1. Mulliken Charge Distribution

**Figure 25:**
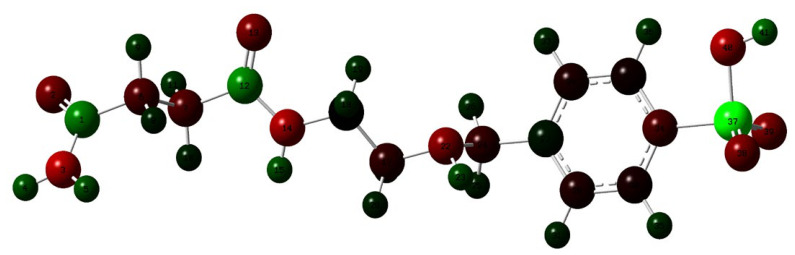
Structure optimization and charge distribution of PASP-S.

The results of Mulliken charge distribution are shown in Figure 25 and Table 3. Here, N, O, and some C atoms in the PASP-S structure are concentrated with negative charges, among which the N of amino group and the O of carboxyl group are enriched with more negative charges. A large number of negative charges can be combined with free Ca^2+^ in the solution. The interaction forms a soluble complex to increase the solubility of calcium scale. In addition, PASP-S can also adsorb on the surface of calcium scale through electrostatic interaction. Thus, the surface of calcium scale crystals is damaged, and the growth of crystal plane is inhibited.

#### 3.9.2. Electric Potential Distribution

**Figure 26:**
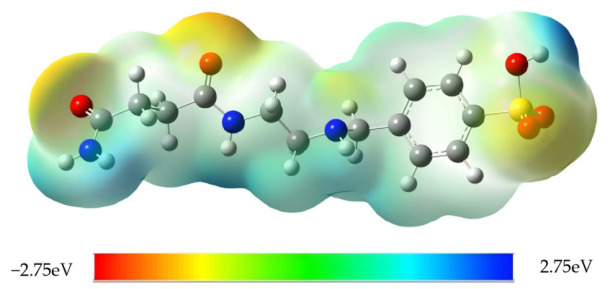
PASP-S electrostatic potential distribution.

Figure 26 shows the electrostatic potential distribution map of the PASP-S molecule, which reflects the internal charge distribution and electrical properties of the molecule. Red and blue represent negative and positive electrostatic potential values respectively. The red regions indicate attraction to positive charges (i.e., negative electrostatic potential), while the blue regions indicate repulsion to positive charges (i.e., positive electrostatic potential). In the PASP-S molecule, negative charges are concentrated near the N and O atoms. These regions can attract free Ca^2+^ ions in the solution and repel scaling anions simultaneously. In addition, positive charges are mainly distributed uniformly along the carbon chain, which can disperse scaling anions evenly. This significantly reduces collisions between scaling ions, thereby enhancing the molecule’s scale inhibition performance and iron oxide dispersion performance.

#### 3.9.3. Frontline Orbital Theory

**Figure 27:**
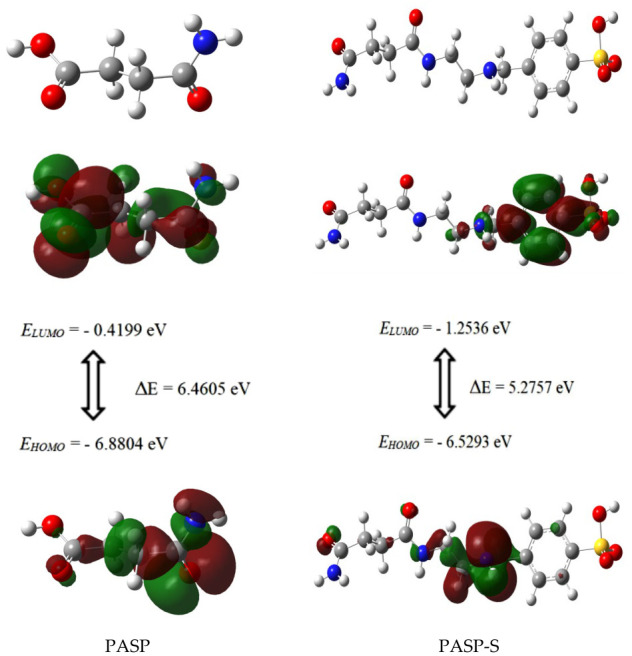
Optimized structures and HOMO and LUMO orbitals of PASP and PASP-S.

Based on the density functional theory (DFT), the relationship between the molecular orbitals of PASP-S and corrosion inhibition performance was investigated. The frontier orbitals of PASP and PASP-S are shown in Figure 27, and the quantum chemical calculation parameters are listed in Table 4. Compared with PASP, PASP-S has a higher EHOMO and lower ELUMO. Furthermore, the smaller ΔE indicates enhanced electron mobility in the PASP-S molecule, enabling easier electron transfer and higher reactivity. Additionally, the lower total electronegativity and hardness demonstrate superior inhibition capability. Notably, the positive ΔN value of PASP-S signifies that it suppresses carbon steel corrosion by transferring electrons to metal surfaces to form an adsorption film. Moreover, the positive ΔN PASP-S is greater than ΔN PASP. This shows that compared with PASP, PASP-S has a stronger ability to provide electrons and is easier to adsorb on the surface of carbon steel.

## 4. Conclusions

(1) A novel water treatment agent, ethylenediamine–benzenesulfonic acid-modified polyaspartic acid (PASP-S), was successfully synthesized using an aminolysis ring-opening reaction by grafting 4-bromomethylbenzenesulfonic acid onto the side chains of PASP using ethylenediamine.

(2) The scale inhibition performance of PASP-S was determined using the static test method. PASP-S exhibits satisfactory scale inhibition effects on CaCO_3_ and CaSO_4_ scales, and presents superior inhibition performance toward Ca_3_(PO_4_)_2_ scale. PASP-S can adsorb onto crystal surfaces to disrupt the regular morphology, occupy growth sites, and restrain crystal plane growth. Meanwhile, it can disperse scale-forming ions and reduce crystal size, and its scale inhibition capacity is remarkably enhanced with the increase in dosage concentration.

(3) PASP-S significantly improves the corrosion inhibition performance of PASP. Through adsorption, it effectively covers and forms a barrier film on the surface of the test piece to prevent the corrosion medium from eroding the surface of the test piece.

(4) PASP-S significantly inhibited the growth of *Escherichia coli* but did not inhibit the growth of *Staphylococcus aureus* and *Bacillus cereus*. This substance has high antibacterial specificity and has the potential to regulate the growth of bacterial colonies in circulating water.

The sulfonated modified polyaspartic acid (PASP-S) synthesized in this study exhibits remarkable advantages over conventional polyaspartic acid (PASP) and commonly used organophosphorus and carboxylic acid scale inhibitors. As a classic green and biodegradable water treatment agent, pristine PASP possesses environmentally friendly characteristics such as non-phosphorus property and biodegradability. By introducing sulfonic acid functional groups via sulfonation modification, PASP-S retains the inherent merits of PASP, including green performance, biodegradability, and phosphorus-free environmental friendliness. Meanwhile, its inhibition capacity against calcium sulfate and calcium phosphate scale is greatly enhanced. This modification breaks through the limitation of pristine PASP, which only shows satisfactory inhibition on calcium carbonate scale with poor adaptability to calcium phosphate scale. The scale inhibition spectrum is significantly broadened, and the corrosion inhibition efficiency is also obviously improved. Compared with phosphorus-containing scale inhibitors such as ATMP, PASP-S is phosphorus-free, pollution-free, and biodegradable. It meets the requirements of green water environment remediation and possesses superior engineering application potential and economic benefits.

## Figures and Tables

**Table 1:** Open circuit potential of carbon steel electrode in 3.5% NaCl solution containing different concentrations of PASP-S.

c/mg/L	*E_corr_*/mV	Δ*E_corr_*/mV	Corrosion Inhibition Type
0	641.25	—	
10	609.34	31.91	Mixed type
30	562.23	79.02	Mixed type
50	562.78	78.47	
70	547.23	94.02	Anodic type
100	551.35	89.90	

**Table 2:** Tafel curve electrochemical polarization parameters.

Inhibitor	*c*	*β*_a_	−*β*_c_	*I*_corr_	*η*	*θ*
	mg/L	mV/dec	mV/dec	mA/cm^2^	%	
PASP-S	0	162.79	84.64	7.586 × 10^−6^	—	—
	10	108.30	83.98	3.930 × 10^−6^	48.19	0.4819
	30	75.54	109.05	2.605 × 10^−6^	65.00	0.6500
	50	109.35	81.06	3.273 × 10^−6^	56.85	0.5685
	70	77.34	109.79	3.314 × 10^−6^	56.31	0.5631
	100	76.30	143.41	4.210 × 10^−6^	44.50	0.4450

**Table 3:** Mulliken charge distribution of PASP-S.

Label	Element	Charge/eV	Label	Element	Charge/eV
1	C	13.7273	22	N	−14.2900
2	O	−12.5182	23	H	7.6318
3	N	−19.4132	24	C	−5.4459
4	H	8.9995	25	H	3.5620
5	H	8.6382	26	H	4.4188
6	C	−8.3372	27	C	2.2785
7	C	−8.5410	28	C	−3.7009
8	H	3.2972	29	C	−3.2890
9	H	5.5541	30	C	−2.6841
10	H	3.2287	31	H	3.8723
11	H	5.3912	32	C	−1.9143
12	C	14.6063	33	H	4.2342
13	O	−12.8715	34	C	−8.2446
14	N	−17.9751	35	H	4.9215
15	H	8.3945	36	H	5.5548
16	C	−0.2231	37	S	33.4614
17	C	−5.0445	38	O	−13.6515
18	H	4.4780	39	O	−13.4342
19	H	4.7568	40	O	−17.9700
20	H	3.4536	41	H	11.2703
21	H	3.8173			

**Table 4:** Quantum chemical calculation parameters of PASP and PASP-S.

Inhibitor	*E_HOMO_*/eV	*E_LUMO_*/eV	Δ*E*/eV	*χ*/eV	*η*/eV	Δ*N*
PASP	−6.8804	−0.4199	6.4605	3.6502	3.2303	0.5185
PASP-S	−6.5293	−1.2536	5.2757	3.8915	2.6379	0.5892

## Data Availability

The original contributions presented in this study are included in the article. Further inquiries can be directed to the corresponding author.
